# Low Na, High K Diet and the Role of Aldosterone in BK-Mediated K Excretion

**DOI:** 10.1371/journal.pone.0115515

**Published:** 2015-01-21

**Authors:** Ryan J. Cornelius, Donghai Wen, Huaqing Li, Yang Yuan, Jun Wang-France, Paige C. Warner, Steven C. Sansom

**Affiliations:** Department of Cellular and Integrative Physiology, University of Nebraska Medical Center, Omaha, Nebraska, United States of America; University of Louisville, UNITED STATES

## Abstract

A low Na, high K diet (LNaHK) is associated with a low rate of cardiovascular (CV) disease in many societies. Part of the benefit of LNaHK relies on its diuretic effects; however, the role of aldosterone (aldo) in the diuresis is not understood. LNaHK mice exhibit an increase in renal K secretion that is dependent on the large, Ca-activated K channel, (BK-α with accessory BK-β4; BK-α/β4). We hypothesized that aldo causes an osmotic diuresis by increasing BK-α/β4-mediated K secretion in LNaHK mice. We found that the plasma aldo concentration (P[aldo]) was elevated by 10-fold in LNaHK mice compared with control diet (Con) mice. We subjected LNaHK mice to either sham surgery (sham), adrenalectomy (ADX) with low aldo replacement (ADX-LA), or ADX with high aldo replacement (ADX-HA). Compared to sham, the urinary flow, K excretion rate, transtubular K gradient (TTKG), and BK-α and BK-β4 expressions, were decreased in ADX-LA, but not different in ADX-HA. BK-β4 knockout (β4KO) and WT mice exhibited similar K clearance and TTKG in the ADX-LA groups; however, in sham and ADX-HA, the K clearance and TTKG of β4KO were less than WT. In response to amiloride treatment, the osmolar clearance was increased in WT Con, decreased in WT LNaHK, and unchanged in β4KO LNaHK. These data show that the high P[aldo] of LNaHK mice is necessary to generate a high rate of BK-α/β4-mediated K secretion, which creates an osmotic diuresis that may contribute to a reduction in CV disease.

## Introduction

Cardiovascular disease and stroke are more prevalent in societies subjected to a “Western” diet, as opposed to the “ancient” or “Mediterranean” diet, which comprises a preponderance of fruits and vegetables and is lower in Na, higher in K and more alkaline [1–3]. However, the low Na, high K, alkaline diet is associated with a high plasma aldosterone (aldo) concentration (P[aldo]), which is known as the end product of the renin-angiotensin system and is often associated with hypertension [4,5]. It is therefore interesting that the low Na, high K diet is associated with low cardiovascular disease [3,6,7].

This laboratory has recently studied the handling of a low Na, high K, alkaline diet (LNaHK) in mice [8,9]. The LNaHK was designed to mimic that of the South American Yanomami, who exhibited very low Na, high K and low Cl concentrations in urinary samples. Little is understood about maintaining electrolyte homeostasis and the role of aldo in animals on LNaHK. Because LNaHK contains a 5% K content, compared with 0.6% K of a control diet (Con), the K must be eliminated at a very rapid rate. However, mice maintain K balance with no change in blood volume and only a slightly elevated plasma [K] (P[K]) [9]. When consuming LNaHK, filtered Na bypasses the Na-Cl-cotransporter (NCC) in the early distal tubule and is delivered for reabsorption to the epithelial Na channel (ENaC) in the connecting tubule (CNT) and the cortical collecting duct (CCD) [10]. Therefore, thiazides, which are effective NCC inhibiting diuretics in Con mice, do not elicit a natriuretic diuresis in LNaHK mice [9].

The beneficial cardiovascular effects of a high K diet may be due to its strong diuretic effect [11,12]. High K intake decreases NaCl reabsorption in the thick ascending limb via the Na-K-Cl-cotransporter (NKCC2) [13] and in the early distal tubule via NCC [14,15]. However, another mechanism should be involved in LNaHK diet diuresis since Na excretion is minimal due to the low NaCl consumption. Moreover, high aldo should enhance Na reabsorption rather than oppose it.

The diuretic effect of LNaHK might rely on aldo-induced K secretion mediated by the renal large, Ca-activated K channels (BK). Activation of BK channels in the distal nephron and vascular smooth muscle has been associated with reduced blood pressure [16–21]. Although the renal outer medullary K channel (ROMK) of CCD principal cells (PC) mediates K secretion in Con animals [22–25], K excretion during high K, alkaline conditions is mediated by BK of intercalated cells (IC), comprised of the BK-α pore and the BK-β4 subunit (BK-α/β4), along with an aldo-induced increase in PC-mediated K excretion [8,9]. Despite its localization in IC, BK-α/β4-mediated K secretion is driven by ENaC-mediated Na reabsorption in PC and may enhance the K secreted to Na reabsorption ratio in the CNT/CCD to a value greater than one [9].

In animals on a regular Na, high K diet (HK) aldo increases the expression of ENaC [26–28] and Na-K-ATPase [29,30] in the CNT and CCD to enhance the Na reabsorption that drives K secretion. The combination of low Na and high K consumption results in both P[K] and angiotensin II stimulating very high levels of aldo production from the adrenal glomerulosa. However, we do not know whether the very high P[aldo] is necessary to increase BK expression, BK-α/β4-mediated K excretion, or urinary flow.

In this study, we determined the role of P[aldo] to maintain BK-α/β4-dependent K homeostasis in LNaHK mice by employing adrenalectomy (ADX) for P[aldo] reduction and osmotic pump infusion for aldo repletion. The P[aldo], which is elevated to greater than 10-fold in mice on LNaHK, maintains K homeostasis, independent of P[K], by increasing BK-α/β4-mediated K secretion, which creates a kaliuretic osmotic diuresis in the aldo-sensitive distal nephron. In contrast to a high Na diet that tends to expand blood volume, the high aldo-induced osmotic diuresis would counteract volume expansion and reduce the probability of developing cardiovascular disease and stroke.

## Results

### Role of aldo in K homeostasis in LNaHK mice

**Figure 1:**
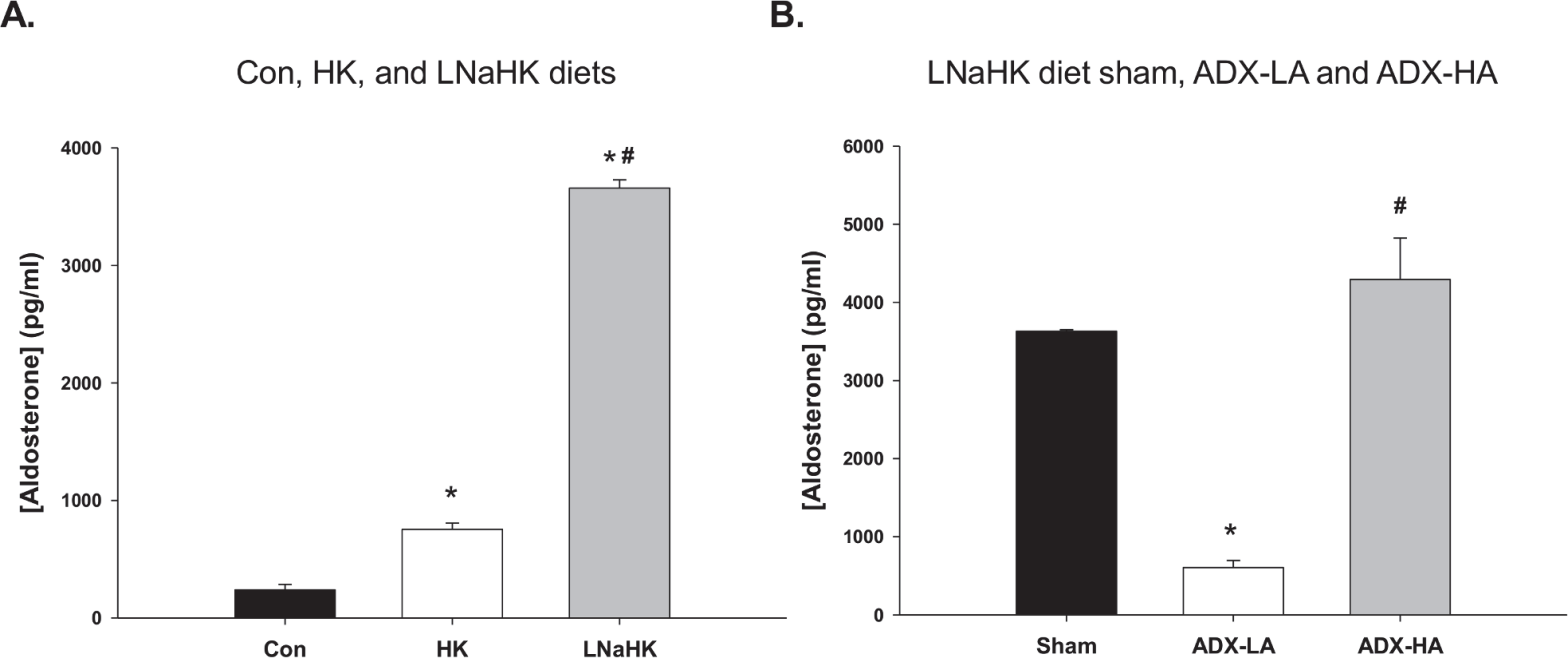
The effect of high K diets, adrenalectomy (ADX), and aldo replacement on plasma aldosterone (aldo) concentration (P[aldo]). **(A)** Bar plot of P[aldo] in sham mice on control (Con; *n* = 5), high K (HK; *n* = 7), and low Na, high K (LNaHK; *n* = 6) diets. * *P* < 0.001 vs. Con; # *P* < 0.001 vs. HK. **(B)** Bar plot of P[aldo] in sham (*n* = 4) and ADX mice receiving a low dose replacement of aldo (ADX-LA; *n* = 6), and ADX mice receiving high dose aldo (ADX-HA; *n* = 7). * *P* < 0.001 vs. sham; # *P* < 0.001 vs. ADX-LA.

We examined the role of aldo, as opposed to high P[K], in maintaining K homeostasis in LNaHK mice. We first compared P[aldo] levels of sham mice that received Con, HK, or LNaHK diet. As shown by the bar plot of Fig. 1A, the P[aldo] of HK had a value of 753.2 ± 54.4 pg/ml, twice the value of Con. The P[aldo] of LNaHK was 15-fold greater than Con levels and more than 4-fold that of HK.

Table 1 compares various plasma and urine values of sham and ADX Con mice. As shown, the P[aldo] of ADX Con mice was reduced to 15% of the sham Con mice. The ADX Con mice exhibited a significant increase in P[K], and significant decreases in transtubular K gradient (TTKG) and K excretion rate (U_K_V). ADX Con mice exhibited minimal but significant reductions in the ability to secrete and eliminate K.

**Table 1:** Characteristics of WT sham and ADX mice on control diet.

	**Sham Con**		**ADX Con**		
**Blood**	**Value**	**SEM**	**Value**	**SEM**	**P value**
[K^+^] (mM)	**3.83**	0.11	**4.48**	0.10	**0.001**
[Na^+^] (mM)	**152**	0.6	**150.2**	0.95	NS
Osmolality (mOsm)	**305**	2.9	**301.7**	3.1	NS
Hct (%)	**43.3**	1.2	**40.5**	2.0	NS
BUN (mg/dL)	**31.8**	2.4	**29.7**	2.0	NS
P[aldo] (pg/ml)	**239.0**	46.4	**55.8**	9.2	**0.002**
**Urine**					
pH	**6.29**	0.21	**6.48**	0.27	NS
Volume (ml/day)	**1.52**	0.23	**0.84**	0.14	**0.03**
[K^+^] (mM)	**310.5**	23.5	**231.9**	18.9	**0.03**
[Na^+^] (mM)	**142.5**	24.7	**176.1**	17.0	NS
Osmolality (mOSM)	**2007**	126	**1858**	169	NS
**K^+^ handling**					
U_K_V (μmol/day)	**457.0**	47.0	**183.9**	19.3	**<0.001**
TTKG	**12.4**	0.7	**8.5**	0.4	**0.016**
**Intake**					
Food (g/day)	**2.80**	0.08	**2.51**	0.24	NS
Water (ml/day)	**3.70**	0.40	**3.40**	0.40	NS

Abbreviations: Con, control; ADX, adrenalectomy; SEM, standard error of mean; NS, not significant; Hct, hematocrit; BUN, blood urea nitrogen; P[aldo]; plasma [aldosterone]; U_K_V, rate of K excretion; TTKG, transtubular K gradient. Sham Con, *n* = 5; ADX Con, *n* = 6.

We determined the effects of ADX in LNaHK mice and whether aldo replacement could restore the capacity of the mice to maintain K homeostasis. We could not obtain urinary or plasma values for ADX mice on LNaHK because these mice expired within 24 h after being placed on LNaHK. However, ADX mice on LNaHK thrived and maintained K homeostasis when receiving a replacement dose of aldo that resulted in a P[aldo] similar to that of the sham on LNaHK (Fig. 1B). ADX mice receiving a low aldo replacement dose that resulted in a P[aldo] of 606.7 pg/ml (Fig. 1B), a value not different from that of sham Con mice (Fig. 1A), survived long enough for analysis of plasma and urinary electrolyte values after four days on LNaHK. Consequently, we compared (Table 2) sham LNaHK mice along with these two groups of aldo replacement mice: ADX LNaHK with partial, low aldo replacement (ADX-LA), and ADX LNaHK with complete, high aldo replacement (ADX-HA).

**Table 2:** Characteristics of WT LNaHK sham, ADX-LA, and ADX-HA mice.

	**Sham**		**ADX-LA**		**ADX-HA**	
**Blood**	**Value**	**SEM**	**Value**	**SEM**	**Value**	**SEM**
[K^+^] (mM)	**4.25**	0.15	**7.40**	0.50	**4.23**	0.52
[Na^+^] (mM)	**147.5**	1.9	**141.2**	0.4	**147.6**	0.6
Osmolality (mOsm)	**296**	3	**292**	4	**304**	2
Hct (%)	**43.4**	1.0	**51.5**	2.0	**48.1**	1.5
BUN (mg/dL)	**23.3**	1.7	**38.2**	5.0	**22.3**	1.1
P[aldo] (pg/ml)	**3629.7**	20.5	**606.7**	89.4	**4292.8**	530.0
**Urine**						
pH	**8.43**	0.24	**8.61**	0.15	**7.83**	0.20
Volume (ml/day)	**6.19**	0.36	**1.43**	0.27	**4.69**	0.33
[K^+^] (mM)	**423.9**	26.5	**719.6**	42.6	**579.5**	29.1
[Na^+^] (mM)	**6.5**	0.6	**12.7**	1.6	**2.9**	0.3
Osmolality (mOsm)	**1005**	69	**1681**	78	**1454**	60
**Intake**						
Food (g/day)	**4.32**	0.55	**1.98**	0.22	**3.21**	0.22
Water (ml/day)	**13.90**	1.72	**3.73**	0.27	**9.32**	0.50

Abbreviations: LNaHK, low Na, high K; ADX-LA, adrenalectomy with low dose aldosterone replacement; ADX-HA, adrenalectomy with high dose aldosterone; SEM, standard error of mean; Hct, hematocrit; BUN, blood urea nitrogen; P[aldo]; plasma [aldosterone]. Sham, *n* = 7; ADX-LA, *n* = 6; ADX-HA, *n* = 7.

**Figure 2:**
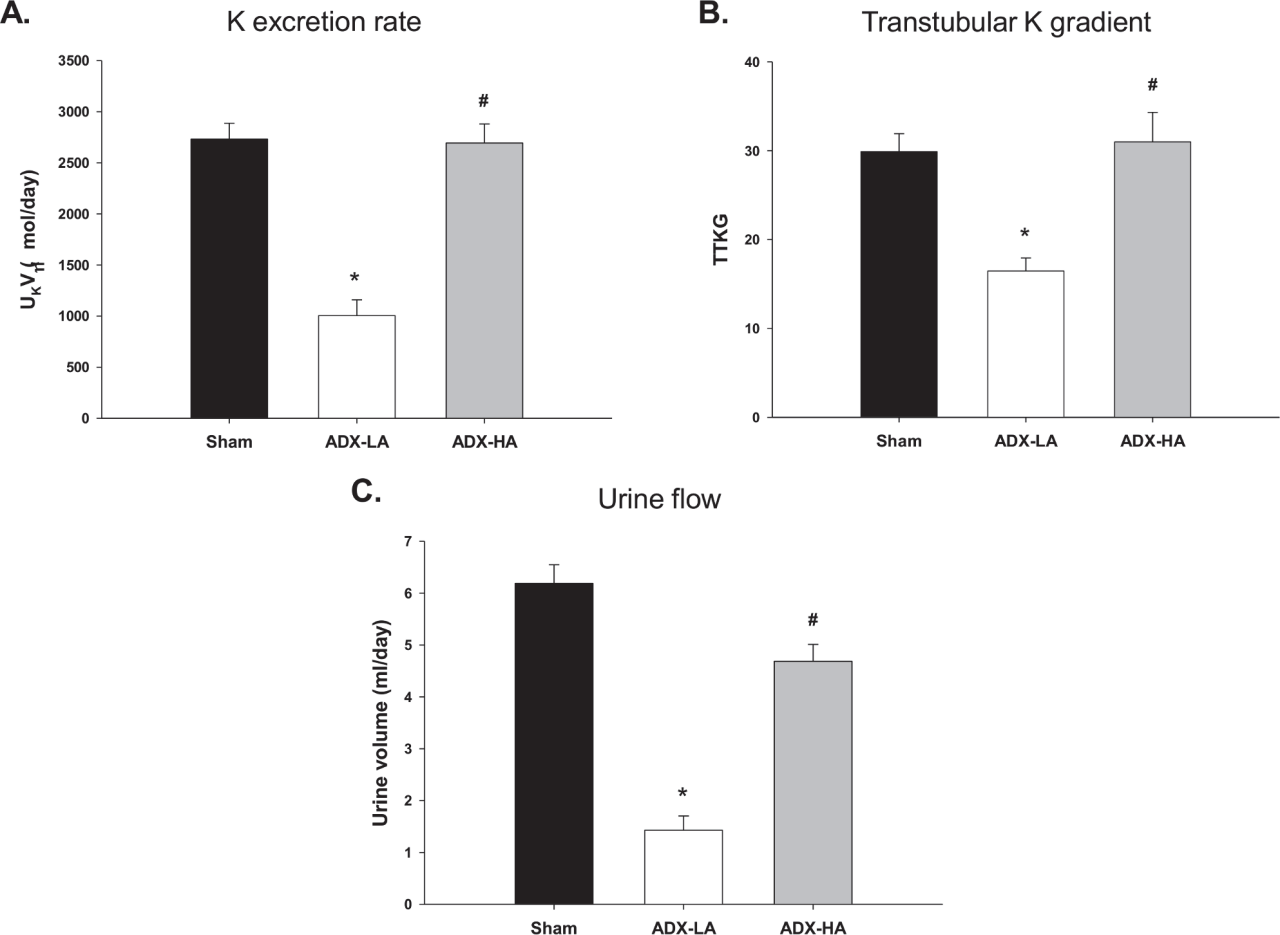
The effects of ADX and aldo replacement on K balance of LNaHK mice. **(A)** The rate of urinary K excretion (U_K_V; * *P* < 0.001 vs. sham; # *P* < 0.001 vs. ADX-LA), **(B)** Transtubular K gradient (TTKG; * *P* = 0.002 vs. sham; ^#^ *P* = 0.002 vs. ADX-LA), and **(C)** Urinary flow (* *P* < 0.001 vs. sham; # *P* < 0.001 vs. ADX-LA) in sham (*n* = 7), ADX-LA (*n* = 6), and ADX-HA (*n* = 7) mice.

The complete replacement dose of aldo, ADX-HA, restored K homeostasis in mice on LNaHK; however, LNaHK mice receiving ADX-LA, demonstrated significant disruption of K homeostasis. Both the U_K_V (Fig. 2A) as well as the TTKG (Fig. 2B) decreased significantly in ADX-LA (1004.7 ± 154.5 μmol/day and 16.5 ± 1.5, *n* = 6) compared with sham (2731.6 ± 155.0 μmol/day and 29.9 ± 2.0, *n* = 7) and ADX-HA (2693.4 ± 186.5 μmol/day and 31.0 ± 3.3, *n* = 7). The U_K_V and TTKG of ADX-HA were not different from sham. The urinary flow of ADX-LA mice decreased compared with sham and ADX-HA, to a value of 1.4 ml/day (Fig. 2C), which was similar to Con mice (Table 1). There was no difference in plasma [creatinine] between sham (0.087 ± 0.005 mg/dl, *n* = 4), ADX-LA (0.118 ± 0.014 mg/dl, *n* = 5), and ADX-HA (0.118 ± 0.008 mg/dl, *n* = 8) mice, a result consistent with the notion that glomerular filtration was not compromised in the ADX-LA group.

### Role of aldo in BK-α/β4 expression

**Figure 3:**
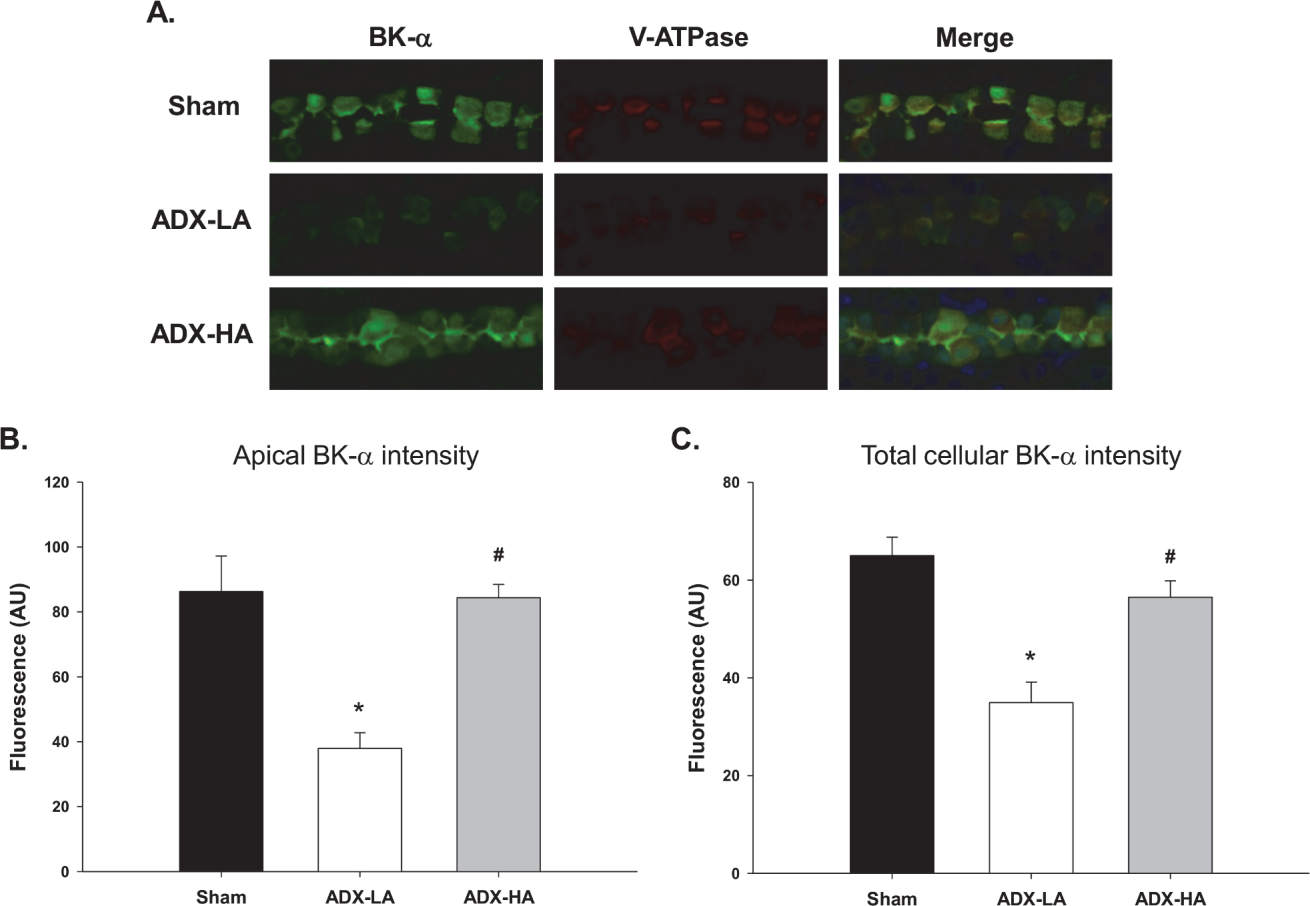
The effect of aldo on BK-α expression in the distal nephron of LNaHK mice. Representative images **(A)** and bar plots summarizing intensity of fluorescent immunohistochemical staining of apical **(B)** and total **(C)** BK-α in cortical kidney sections of sham, ADX-LA, and ADX-HA mice. BK-α (green) was co-stained with V-ATPase (red), a marker of intercalated cells in the connecting tubule and collecting duct. Merged images also contain Hoechst nuclear stain (blue). * *P* < 0.001 vs. sham; # *P* < 0.001 vs. ADX-LA; *n* = 9.

We showed previously that BK-α expression is increased in HK mice [31]. Fig. 3 illustrates the staining intensities of sham, ADX-LA and ADX-HA. As shown by the representative staining (Fig. 3A) and the summary bar plots (Fig. 3B and 3C), both apical and total cellular BK-α expression of ADX-LA were decreased compared to sham. BK-α expression of ADX-HA was not different from sham, and was increased compared to ADX-LA.

**Figure 4:**
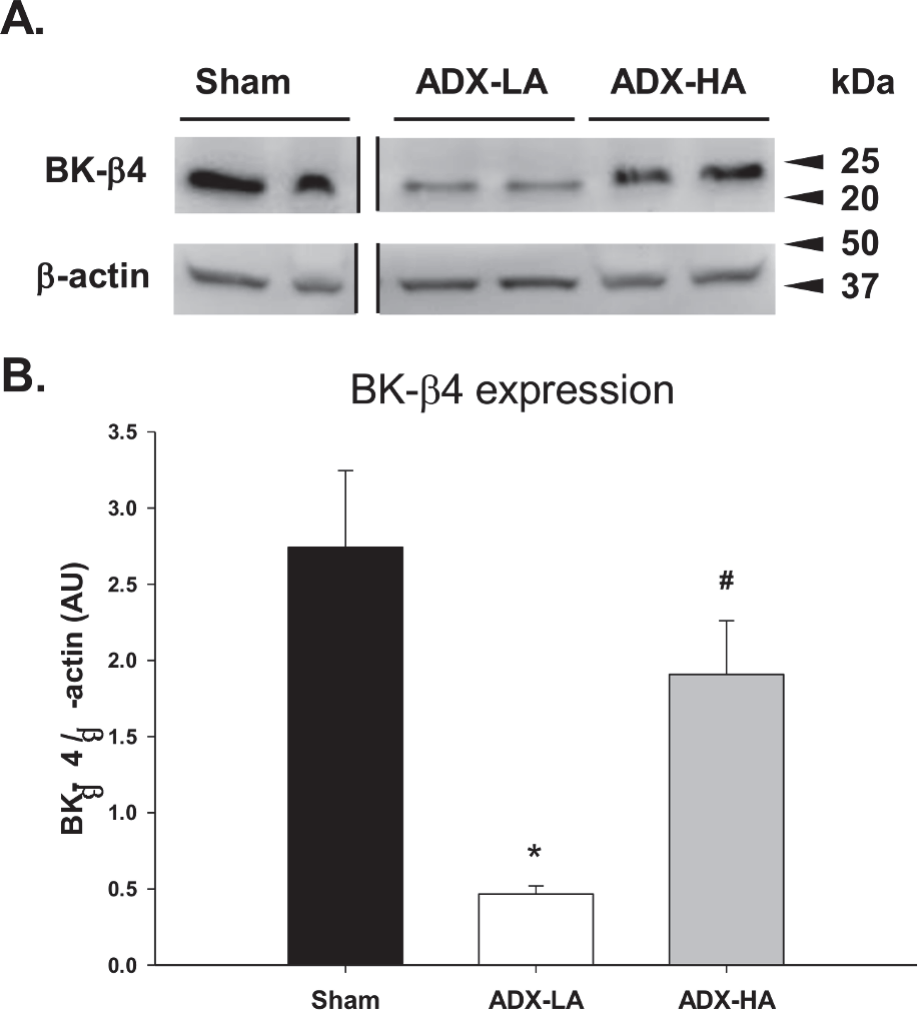
The effect of aldo on BK-β4 expression of LNaHK mice. Western blot representative immunoblots **(A)** and summary bar plots **(B)** of BK-β4 expression in kidney cortex of sham, ADX-LA, and ADX-HA mice. β-actin was used as a loading control. * *P* = 0.004 vs. sham; # *P* = 0.019 vs. ADX-LA; *n* = 4. All bands shown were from the same blot.

Fig. 4 illustrates the BK-β4 protein expression as measured by Western blot. The representative immunoblots (Fig. 4A) and densitometry analysis (Fig. 4B) demonstrated that BK-β4 expression decreased in ADX-LA mice compared to sham. BK-β4 expression of ADX-HA mice was increased compared to ADX-LA, but was not different from sham.

### Role of aldo in BK-α/β4-mediated K secretion

**Figure 5:**
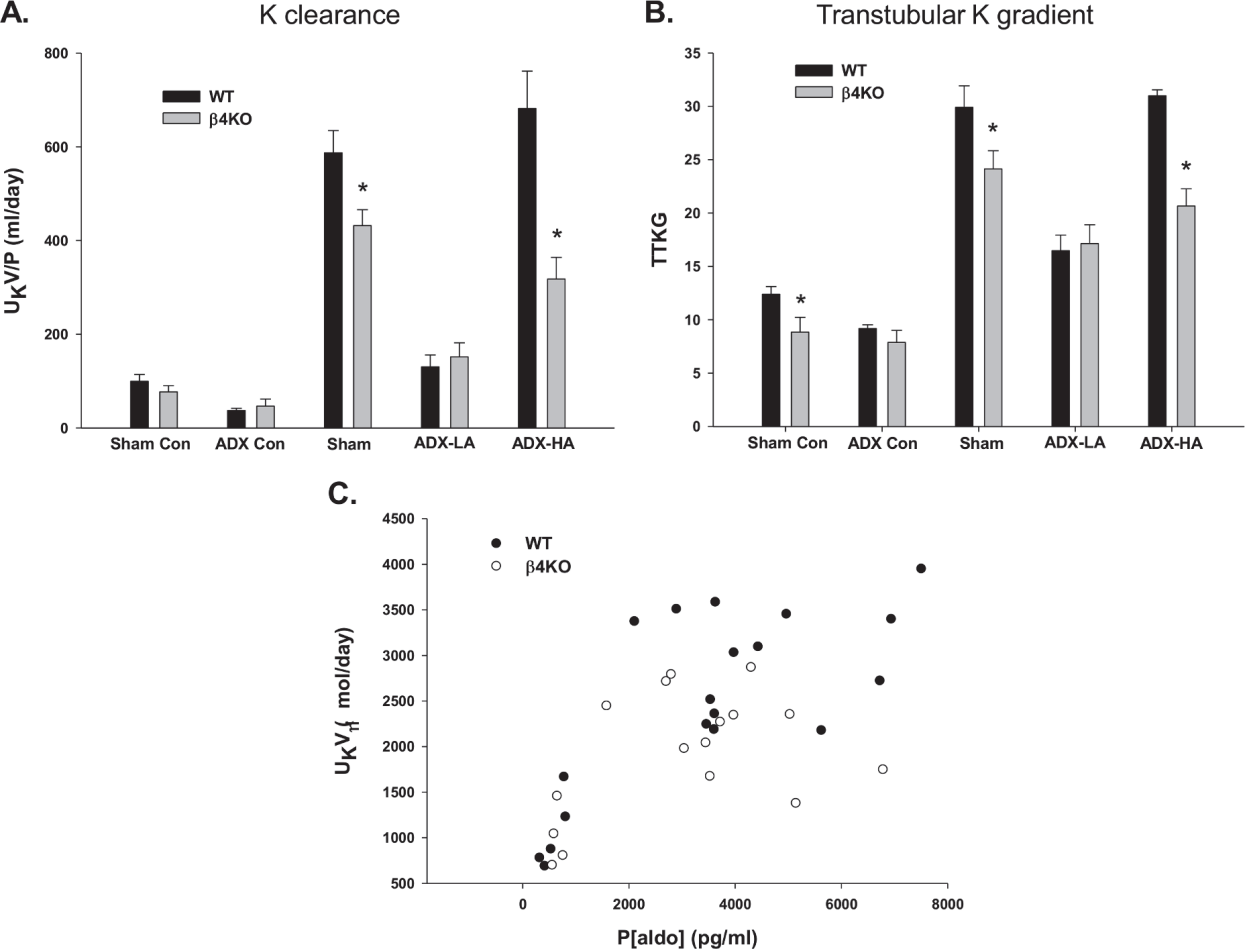
The effect of aldo on β4KO LNaHK mice. The K clearance **(A)** and TTKG **(B)** of WT and β4KO mice on Con given sham or ADX surgery or on LNaHK given sham, ADX-LA, or ADX-HA. * *P* < 0.05 vs. WT. **(C)** The relation between P[aldo] and U_K_V determined in WT and β4KO ADX mice receiving various doses of aldo replacement.

We showed previously that the apical BK-α expression is increased by the presence of the BK-β4 subunit in IC of the mouse distal nephron and that BK-α/β4-mediated K secretion is attenuated in the absence of BK-β4 [31]. Here we determined aldo regulation on BK-α/β4-mediated K excretion by comparing K handling of WT with BK-β4 knockout mice (β4KO), which received the same experimental treatment as the WT mice described above. The various plasma and urinary values for WT and β4KO are reported in Table 2 and Table 3, respectively. The K excretion of LNaHK sham, ADX-LA, and ADX-HA WT and β4KO mice are shown in Fig. 5. The K clearance (Fig. 5A) and TTKG (Fig. 5B) were considerably reduced in LNaHK sham β4KO (431.9 ± 33.8 ml/day, *n = 7* and 24.1 ± 1.7, *n* = 7) compared with WT (587.4 ± 47.2 ml/day, *n* = 10 and 29.9 ± 2.0, *n* = 7). Similarly, in the ADX-HA groups, the K clearance (317.9 ± 46.2 ml/day, *n* = 5) and TTKG (20.7 ± 1.6, *n* = 5) of β4KO were significantly less compared with WT (681.8 ± 79.6 ml/day, *n* = 7 and 31.0 ± 0.6, *n* = 11). However, we observed no difference in K clearance and TTKG between WT (130.4 ± 25.1 ml/day and 16.5 ± 1.5, *n* = 4) and β4KO (151.6 ± 30.1 ml/day and 17.1 ± 1.8, *n* = 5) in the ADX-LA groups. These results indicate that BK-α/β4 mediates K secretion in mice given LNaHK only when P[aldo] is increased to the high levels shown in Fig. 1.

**Table 3:** Characteristics of β4KO LNaHK sham, ADX-LA, and ADX-HA mice.

	**Sham**		**ADX-LA**		**ADX-HA**	
**Blood**	**Value**	**SEM**	**Value**	**SEM**	**Value**	**SEM**
[K^+^] (mM)	**5.17**	0.34	**7.02**	0.68	**6.46**	0.27
[Na^+^] (mM)	**147.4**	1.2	**147.2**	0.7	**149.4**	0.8
Osmolality (mOsm)	**290**	7	**300**	0	**304**	3
Hct (%)	**43.3**	1.1	**45.0**	2.1	**43.6**	1.6
BUN (mg/dL)	**21.4**	1.0	**40.3**	4.4	**26.6**	0.9
P[aldo] (pg/ml)	**2771.9**	277.7	**554.4**	84.7	**3656.0**	352.0
**Urine**						
pH	**8.51**	0.17	**7.43**	0.41	**7.65**	0.35
Volume (ml/day)	**5.36**	0.39	**1.42**	0.30	**2.43**	0.17
[K^+^] (mM)	**431.8**	24.0	**743.4**	63.4	**826.0**	89.8
[Na^+^] (mM)	**5.0**	0.9	**14.8**	2.3	**8.6**	1.1
Osmolality (mOsm)	**1027**	48	**1932**	126	**1867**	258
**Intake**						
Food (g/day)	**3.22**	0.31	**1.56**	0.09	**1.69**	0.21
Water (g/day)	**7.81**	1.12	**4.25**	0.22	**5.39**	0.42

Abbreviations: LNaHK, low Na, high K; ADX-LA, adrenalectomy with low dose aldosterone replacement; ADX-HA, adrenalectomy with high dose aldosterone; SEM, standard error of mean; Hct, hematocrit; BUN, blood urea nitrogen; P[aldo]; plasma [aldosterone]. Sham, *n* = 7; ADX-LA, *n* = 5; ADX-HA, *n* = 5.

Fig. 5C shows the relation between U_K_V and P[aldo] of WT and β4KO mice receiving various doses of aldo replacement. As shown, WT and β4KO U_K_V increased steeply at a P[aldo] of ~500 pg/ml to 3,000 pg/ml; however, the U_K_V leveled and did not increase additionally between the values of 3,000 to 8,000 pg/ml. The U_K_V of β4KO (2022.4 ± 270.6 μmol/day, *n* = 5) was lower (*P* = 0.035) than WT (2731.6 ± 155.0 μmol/day, *n* = 7) mice on LNaHK.

### Amiloride-sensitive osmotic diuresis

Mice on LNaHK [9], or HK [8,32,33] exhibit a urinary flow that is 5-fold that of Con mice. A high urine volume is necessary to dilute the K in the CNT/CCD and excrete K at a high rate because the concentration of K that can be secreted in the CNT/CCD is limited to approximately 120 mM [9].

The present study attempted to answer whether high urinary flow was the result of aldo—induced high rate of K secretion in the CNT/CCD, thereby creating a high K osmotic gradient that would oppose the extraction of water via aquaporin 2 stimulated by ADH. If the secreted distal [K] is responsible for a high osmotic diuresis in the LNaHK mice then the osmolar clearance (COsm) should be decreased by amiloride, which inhibits the Na reabsorption for K secretion exchange in the CNT/CCD.

**Figure 6:**
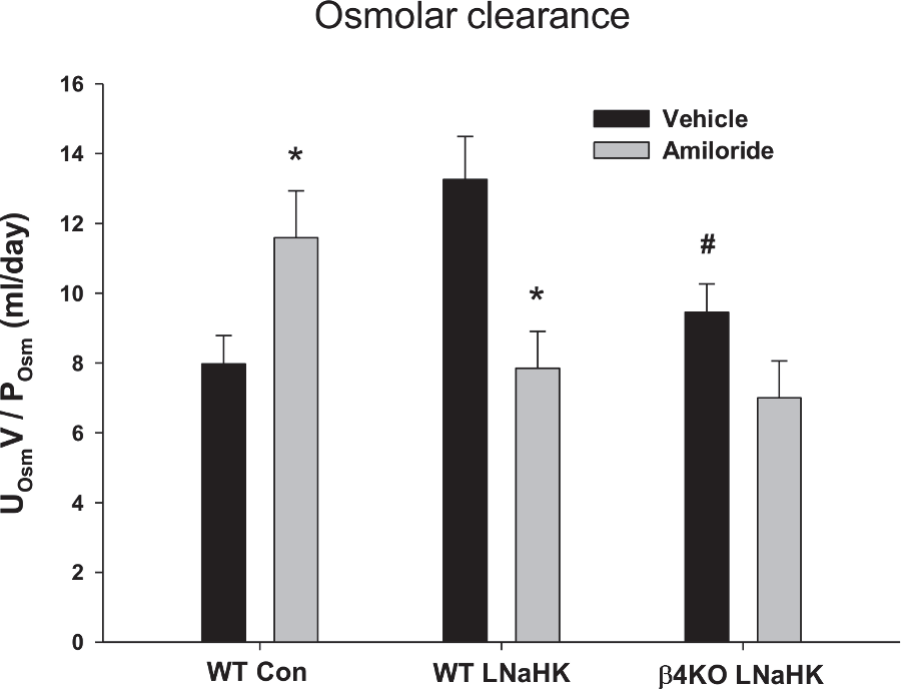
The effects of amiloride on osmolar clearance. The osmolar clearance was determined in WT Con (*n* ≥ 5), WT LNaHK (*n* = 9), and β4KO LNaHK (*n* ≥ 5) mice given vehicle or amiloride. * *P* < 0.05 vs. vehicle; # *P* < 0.05 vs. WT LNaHK.

Fig. 6 shows the COsm for WT Con, WT LNaHK, and β4KO LNaHK mice with vehicle or amiloride treatment. Compared to vehicle, amiloride treatment caused COsm to increase in WT Con (vehicle = 8.0 ± 0.8 ml/day, *n* = 6; amiloride = 11.6 ± 1.3 ml/day, *n* = 5) and decrease in WT LNaHK (vehicle = 13.3 ± 1.2 ml/day, *n* = 9; amiloride = 7.8 ± 1.1 ml/day, *n* = 9), while β4KO LNaHK mice remained unchanged (vehicle = 9.5 ± 0.8 ml/day, *n* = 9; amiloride = 7.0 ± 1.1 ml/day, *n* = 5). These results are consistent with an aldo-enhanced osmotic diuresis as part of the mechanism for the increased urine flow.

## Discussion

A diet comprised of low Na and high K has been touted by cardiovascular researchers and health experts for many years. The “Paleo” diet, as well as vegetarian or vegan diets, is a variation of the LNaHK. A dietary plan was developed by the The National Heart, Lung, and Blood Institute and approved by the USDA and called Dietary Approaches to Stop Hypertension (DASH). The DASH diet, shown to decrease blood pressure and risk of cardiovascular disease, emphasizes eating K rich foods (4,700 mg) and limiting Na consumption to 1,500 mg per day.

The LNaHK diet is an extreme model of the “Paleo” diet [1–3], but is probably similar to the diet of the Yanomami [6,7]. Although the mechanism is still uncertain, high K diets are known as natural diuretics [11,12] and therefore would tend to lower the long-term mean arterial pressure. This study determined the mechanism by which high aldo levels of LNaHK mice induced a diuresis, despite the normal role of aldo to retain Na.

The P[aldo] is elevated in mice on LNaHK to levels 15 times greater than Con mice (Fig. 1A). ADX Con mice exhibited a mild deficiency in K handling (Table 1). However, on LNaHK, ADX mice did not survive without aldo repletion and survived only six days with partial aldo repletion (ADX-LA). The rate of K excretion of LNaHK mice with ADX and complete aldo repletion (ADX-HA) was equal to sham (Fig. 2). The expressions of BK-α and BK-β4 were directly related to the P[aldo] achieved by repletion. β4KO mice with ADX-HA exhibited diminished K clearance, compared with WT (Fig. 5). Amiloride, which prevents Na reabsorption dependent K secretion in the CNT/CCD, increased COsm in Con mice but diminished COsm in WT LNaHK (Fig. 6).

### Influence of diet on P[aldo]

We initially measured P[aldo] of mice on three different diets: Con, HK, and LNaHK using ELISA (Fig. 1A). The P[aldo] for Con mice of 239 pg/ml (Table 1) using ELISA was close to the values of 322 pg/ml [17] and 400 pg/ml [34] of previous studies using radioimmunoassay (RIA). For HK diet, our value of 753 pg/ml using ELISA was less than the value of 1797 ± 630 using RIA [32]; however, the SEM of the RIA value indicates some readings in the range of our value.

Our study showed that mice consuming a diet of LNaHK exhibited a very high P[aldo] that was greater than 3,000 pg/ml (Fig. 1, Table 2). This value is much greater than the value of 1369 pg/ml detected in another study of mice on another low Na, high K diet [35]. However, the dietary K content in that study was 2%, compared with the 5% of our study.

Our results show that the P[aldo] levels of the ADX-HA group were necessary to achieve the high rate of K excretion in the LNaHK mice. When the P[aldo] of ADX mice on LNaHK were partially replete (ADX-LA), with a value of 606 pg/ml (Fig. 1B, Table 2), the mice were unable to achieve a substantial rate of K excretion and the P[K] increased to lethal levels within one week.

### Aldo-dependent BK-α/β4-mediated K secretion

Aldo is the primary hormone regulating K secretion. The mechanism involves increasing Na reabsorption via ENaC [36] in exchange for K secretion via ROMK [37] and BK [38] channels in the CNT/CCD. An increase in P[K], independent of aldo, could regulate the transporters that mediate K secretion. In ADX HK mice, P[K], independent of P[aldo], increased Na-K-ATPase by 200% [39] and increased urine flow and K excretion [40]. K secretion was reportedly independent of ENaC-mediated Na reabsorption in rats with a high K intake, suggesting an aldo-independent mechanism that increases K secretion [41]. However, our results here demonstrate that K secretion in LNaHK mice is completely aldo-dependent.

To establish aldo-independent K secretion it is important that the ADX is complete, because residual adrenal glomerulosa cells can hypertrophy in response to ACTH and secrete substantial aldo [42]. Using ELISA, Con ADX mice exhibited a P[aldo] of 56 pg/ml (Table 1), which is the same value for ADX mice using RIA in a previous study [34]. We were unable to determine the P[aldo] of ADX LNaHK mice because they did not survive for two days without restoring the P[aldo] to at least Con levels. Potassium excretion in mice on ADX-LA, which exhibits near Con levels of P[aldo], is probably mediated by ROMK [10]. Moreover, the ADX-LA mice had P[K] > 7 at 4 days and survived for only 6 days. Therefore, the high rate of K elimination of LNaHK mice is dependent on high levels of P[aldo].

If there was a mechanism to enhance K secretion, independent of aldo-induced Na reabsorption, it would be manifested by LNaHK fed animals, which exhibit minimal distal Na delivery and a very high demand for K secretion. However, the results of our study did not support an aldo-independent mechanism for K secretion in mice on LNaHK. Because aldo regulates Na reabsorption via ENaC in the distal nephron, our result is consistent with a previous study that could not demonstrate Na-independent K secretion in mice on LNaHK [9]. It is possible that there is aldo-independent K excretion in mice on LNaHK but this mechanism is inadequate for survival.

The very high LNaHK P[aldo], which is 4-fold more than the P[aldo] of HK mice, may be necessary to ensure all Na delivery to ENaC. Thiazide diuretics, which inhibit the Na reabsorbing NCC, are ineffective in LNaHK mice because the NCC is inhibited and all Na is delivered to the CNT/CCD for ENaC-mediated Na for K exchange [9,10]. Although aldo enhances NCC-mediated Na reabsorption [43,44], a very high P[aldo] in LNaHK mice may be required to inhibit Na reabsorption via the NCC and maximize Na delivery to ENaC. A paradoxical effect on the NCC for aldo at higher plasma concentrations has not been reported; however, it may involve a non-genomic effect that rapidly activates signaling pathways via an unidentified receptor [45].

There was a difference in the K excretion rate between WT and β4KO on ADX-HA, but no difference between genotypes on ADX-LA (Fig. 5), indicating that the high P[aldo], and not the P[K], is enhancing BK-α/β4-mediated K excretion. An aldo-induced increase in BK activity is consistent with an early study showing that isolated CCDs from mineralocorticoid treated rabbits exhibited an elevated K conductance [46], and a more recent study showing that a HK diet caused increased BK-α expression in IC [31].

The present study shows that the high levels of P[aldo], and not an elevation of P[K], causes the increase in BK-α expression. In our previous study, the effects of spironolactone, an aldo antagonist, did not affect the expression of BK-β4 in HK mice [31]. However, the present study shows that BK-β4 expression is enhanced by aldo in LNaHK mice. Thus, the very high P[aldo] of the LNaHK diet is necessary for the enhanced expression of BK-β4. BK-β4 functions to prevent the lysosomal breakdown of BK-α thereby maintaining its presence in the apical membrane [31].

### Aldo-induced increase in urine flow

At least two mechanisms are involved in the high urinary flow associated with high K diets. With a high K intake, high urinary flow is increased by medullary K recycling, which inhibits the thick ascending limb NaCl reabsorption and the concentrating mechanism [13]. In addition, we have shown here that the high urine flow of LNaHK mice is associated with a high P[aldo], which increased an amiloride-sensitive COsm, indicating a K secreted per Na reabsorption ratio greater than 1. The glomerular filtration rate may also be a factor in diuresis in LNaHK mice. An increased GFR due to either the LNaHK diet or the increased aldo could lead to increased urine flow. The GFR in LNaHK cannot be ruled out as a partial cause for an increase in urine flow.

ADX-LA mice had a food and water consumption (Table 2) similar to Control diet mice but reduced compared to sham LNaHK and ADX-HA. It is unlikely that ADX-LA mice had a reduced urine flow due to an inability to drink water rather than an aldo-dependent decrease in K secretion as the primary defect. If ADX-LA mice drank less water as the primary defect of less P[aldo], then they would be volume depleted with an increased hematocrit. However, the hematocrit levels for ADX-LA were not different compared to sham and ADX-HA (Table 2), indicating similar blood volumes. Therefore, the reduced food and water intake of ADX-LA was probably an adaptation to the increasing P[K] resulting in impaired diuresis.

Our finding that increased flow in mice on LNaHK was aldo dependent is consistent with a recent study examining the handling of a high (5%) K diet in mice with a knockout of aldo synthase (AS-/-). In this study, flow increased by nearly three-fold in WT, but remained the same in AS-/- on a 5% K diet [47].

This relation between P[aldo] and high osmotic distal flow seemingly contradicts a previous study showing that acute aldo infusion to ADX rats does not increase K excretion because aldo stimulates Na reabsorption in the distal tubule, thereby decreasing urine flow [40]. However, these were acute aldo infusions whereas we treated with aldo for several days. It is also important to distinguish between treating with aldo alone and giving aldo with high K as we did in the present study. We recently learned that high K intake turns off the NCC [14,15]. Thus, giving aldo without K reduces P[K] and favors reabsorption of Na via NCC while reducing ENaC-mediated Na reabsorptive exchange for K. Consistent with our results, KCl infusion alone increased P[K], urine flow, and K excretion [40]. Increasing evidence indicates that the WNK4-SPAK signaling pathway can distinguish between high P[aldo] associated with high and low K intake [48].

Although the rate of K excretion was normalized, ADX-HA mice exhibited a greater urine osmolality and a 30% lower urinary volume compared with sham (Table 2). The failure to correct water output was probably due to the lack of glucocorticoid hormone in the ADX mice. Glucocorticoid hormone enhances water excretion and decreases urine osmolality by inhibiting urea transporters (UT-A1) in the inner medullary tip, thereby inhibiting the concentrating mechanism [49,50].

### Limitations and assumptions

We determined that the K secretion in mice on LNaHK was creating an osmotic diuresis. This conclusion was based on the assumption that K, a non-reabsorbable ion, influences water reabsorption in the collecting duct. However, when inhibiting vasopressin receptors (V2) with tolvaptan, water reabsorption is inhibited without affecting Na reabsorption [51]. In this case, Na is permeable and reabsorbed actively to a very low concentration, since the Na retention is regulated by aldo, without the influence of the diuretic effect of non-reabsorbing water. Inhibiting Na reabsorption with amiloride, in this case, would not influence water transport, which is already inhibited. However, water reabsorption in the collecting ducts depends on a combination of the water permeability and the gradient of the non-permeable solute driving force. We assume the water channels are in the apical (AQP2) and basolateral (AQP3) membranes of the CNT and collecting ducts because the urine is concentrating to approximately 2,000 mOsm. In this case, the non-reabsorbable ions, such as Na or secreted K in the lumen (here due to amiloride inhibition) will create an osmotic force to prevent water transport from the lumen. This is the best explanation for the osmotic diuresis created by amiloride in mice on a normal diet and the decrease in osmotic diuresis when mice on LNaHK are treated with amiloride.

The accuracy of the TTKG may be questionable because there is probably K reabsorption in the collecting ducts. However, as shown in a previous study, the reabsorption of urea in this segment will offset the K flux thereby preserving the accuracy of the equation [9].

### Conclusions

The P[aldo] levels of low Na diet mice result in antidiuresis and Na retention. However, the very high P[aldo] levels of LNaHK mice serve to enhance urinary volume increasing BK-α/β4-mediated K secretion, which creates an osmotic diuresis. The high urinary flow created by BK-α/β4-mediated K secretion could activate more BK-α/β4 in a positive feedback manner or activate the BK-α/β1 of PC, which have flow detecting primary cilia.

## Methods

### Animal studies

The protocol was approved by and all animals were maintained in strict accordance with the Institutional Animal Care and Use Committee of the University of Nebraska Medical Center (protocol #’s: 09-050-07, 11-090-11-FC). All surgery was performed under anesthesia, and all efforts were made to minimize suffering. C57BL/6 (8–12 weeks, Charles River, Wilmington, MA) and β4KO mice (12–20 weeks, generously provided by R. Brenner) were housed in the UNMC animal care facility and maintained on a 12-h day-night cycle, with access to food and water at all times. Mice underwent sham or ADX surgery and were given regular mouse chow (control; 0.3% Na, 0.6% K), high K (#TD.07278; 0.3% Na, 5.0% K) or a low Na, high K (#TD.08240; 0.01% Na, 5.0% K) pellet diet (Harlan Teklad, Madison, WI). The low Na, high K diet contained a mix of carbonate, citrate, and Cl as the counter anions to K. ADX mice received aldo replacement by osmotic pump, implanted subcutaneously, either at a low (25 μg/kg/day) or high (500 μg/kg/day) dose, and recovered for 3 days before being placed on LNaHK for 4–10 days. The scatterplot data in fig. 5 included another group of WT mice that received a larger dose of aldosterone replacement (600 μg/kg/day). Another group of mice were treated with an intraperitoneal bolus of vehicle or amiloride (5 mg/kg), at a concentration of 1 mg/ml, twelve hours before sacrifice. Aldo and amiloride were dissolved in polyethylene glycol. P[aldo] was measured using a competitive ELISA kit (DRG International, Springfield, NJ).

During the last two days of treatment, mice were placed in metabolic cages to record food and water consumption and urine volume in a 24 hr period. Urinary pH was measured and [Na] and [K] were determined using a flame photometer (Jenway Clinical PFP7) as previously described [16]. Urine osmolality was measured with a Model 3250 osmometer (Advanced Instruments). Mice were anesthetized by a ketamine/xylazine mixture and sacrificed by severing the carotid artery. The blood was extracted from the carotid artery and used for measurement of hematocrit and [K] and [Na] using an i-STAT 1 blood gas analyzer (Abbott Laboratories). Excess blood was centrifuged for measurement of plasma osmolality.

The TTKG is a calculation that estimates the K gradient across the final CCD before water is reabsorbed in the medullary collecting duct, concentrating K [9]. TTKG was calculated using the following formula: U_[K]_ X P_Osm_ / P_[K]_ X U_Osm_. The COsm was calculated using the following formula:, U_Osm_ X U_V_ / P_Osm_.

### Immunohistochemical staining

Kidneys were removed post-sacrifice and placed in Histochoice MB (Electron Microscopy Sciences, Hatfield, PA), embedded in paraffin and sectioned onto slides for immunofluorescent staining as previously performed [52]. Primary antibodies were applied overnight as follows: BK-α (mouse monoclonal, diluted 1:200, Neuromab), and V-ATPase (goat polyclonal, diluted 1:200, Santa Cruz). Fluorescent secondary antibodies (donkey anti-mouse IgG conjugated Alexa Fluor 488 and donkey anti-goat IgG conjugated Alexa Fluor 594, diluted 1:200, Intitrogen) were then applied for 1 h. Hoechst stain was used to identify nuclei. Quantification of BK-α signal intensity was determined in single-channel, gray scale images after background correction as performed previously [53]. Each group contained a kidney section from 3 different mice. Three different tubules per kidney section were used to quantify staining intensity.

### Western blotting

Kidney cortex was separated from medulla tissue and used for Western blotting as performed following manufacturer’s protocol (Bio-Rad Laboratories, Hercules, CA) and as performed previously by our lab [53,54]. Primary antibodies used were anti-BK-β4 (rabbit polyclonal, diluted 1:500, Alomone Laboratories), and β-actin (mouse monoclonal, diluted 1:500, Santa Cruz Biotechnology). Secondary antibodies included goat anti-mouse IgG, and goat anti-rabbit IgG-conjugated horseradish peroxidase (diluted 1:30,000, Santa Cruz Biotechnology). Detection of protein was performed using enhanced chemiluminescence (Thermo Scientific, Rockford, IL). Densitometry analysis was performed using imageJ software.

### Plasma [creatinine]

Plasma [creatinine] was measured using high-performance liquid chromatography (HPLC) as described by Dunn et al [55]. Briefly, creatinine was extracted from the samples by mixing with acetonitrile acidified with glacial acetic acid (ACN:GAA) at a 1:4 ratio. The mixture was vortexed thoroughly and kept at −20°C for 15 min before being centrifuged at 10,000 rpm for 10 min at 4°C. The supernatant was transferred to a clean tube and the excess ACN:GAA was evaporated using a speed-vac. Mobile phase was added to the remaining residue containing the creatinine. The instrument (Agilent LC 1200; Santa Clara, CA) was connected to a G1321A Agilent fluorescent detector and the chromatograms were recorded using Agilent ChemStation software. The reversed-phase separations were performed by means of a C_18_ column (Waters Spherisorb 10.0 mm) as the stationary phase which was kept at 45°C. The mobile phase (filtered through a 22 μm nylon filter) consisted of 5 mM Na acetate, with 40 ml/L of methanol, and 10 ml/L of ACN:GAA and was pumped at a constant flow-rate of 1 ml/min. The retention time of creatinine was 1.1 min and was separated from the rest of the compounds present in the plasma in chromatographs obtained at 225 nm.

### Statistical analysis

The data are presented as means ± SEM. Statistical analyses were performed using one-way ANOVA with Student-Newman-Keuls post hoc analysis (*P* < 0.05 considered significant). We performed data management and statistical analyses using SigmaPlot version 11.0.
